# Limitations and Blind Spots of Diffusion-Weighted Imaging in the Evaluation of Acute Brain Ischemia: A Narrative Review

**DOI:** 10.3390/jcm15020885

**Published:** 2026-01-21

**Authors:** Ioannis Nikolakakis, Ioanna Koutroulou, Michail Mantatzis, Stefanos Finitsis, Nikolaos Grigoriadis, Theodoros Karapanayiotides

**Affiliations:** 1 Second Department of Neurology, School of Medicine, Aristotle University of Thessaloniki, AHEPA University Hospital, 54636 Thessaloniki, Greece; yiannisnikolakakis@gmail.com (I.N.); ngrigoriadis@auth.gr (N.G.); tkarapanayiotides@auth.gr (T.K.); 2 Department of Radiology, School of Medicine, Aristotle University of Thessaloniki, AHEPA University Hospital, 54636 Thessaloniki, Greece; mantatzis@gmail.com (M.M.); stefanosfin@gmail.com (S.F.)

**Keywords:** DWI-negative stroke, neuroimaging, stroke, DWI limitations, DWI-Reversal

## Abstract

Diffusion-weighted imaging (DWI) has been increasingly utilized in the emergent evaluation of acute ischemic stroke (AIS) patients. DWI enhances sensitivity and specificity and enables the use of delayed reperfusion treatments in selected cases. However, DWI is not devoid of limitations. DWI-negative AIS is not uncommon in clinical practice and is reported in up to 1 of 4 AIS patients. We reviewed the relevant literature and searched the PubMed and Google Scholar databases for studies reporting on DWI-negative AIS prevalence during the 2021–2025 time period. Additionally, we included cases from our practice to highlight key points. DWI-negative AIS prevalence was 16% in one meta-analysis and ranged from 6.9% to 23.2% in identified studies that met our inclusion criteria. The biological, pathophysiological, technical, epidemiological and clinical factors that contribute to DWI-negative stroke are presented in detail. Overall, the application of diffusion imaging modalities for stroke is not bereft of blind spots despite enhanced sensitivity. Over-reliance on advanced neuroimaging and unfamiliarity with its limitations predispose DWI to errors in AIS assessment. Awareness of the predisposing factors, treatment effect, and prognosis guides appropriate decision-making, promoting good outcomes. Prospective appropriately designed trials should address the lingering questions identified, such as the association between time of imaging and DWI negativity.

## 1. Introduction

Stroke inflicts great socio-economic burden, which is amplified in low- and middle-income countries. In high-income countries, stroke incidence in younger adults has risen and overall stroke prevalence is expected to increase in concert with population growth and aging [1]. Acute reperfusion treatments constitute milestones in acute ischemic stroke management and their application is guided by standard and in selected cases advanced neuroimaging modalities. Non-contrast computed tomography (NCCT) has historically been used to exclude the presence of intracranial hemorrhage; a paradigm shift is currently underway, with healthcare systems switching to magnetic resonance (MR) modalities for the initial evaluation of acute ischemic stroke (AIS) in emergency settings [2]. This shift is supported by the capacity of MR-based protocols to confirm AIS, with a diagnostic accuracy reaching 97.5% [3]. DWI was initially applied in the study of central nervous system tumors and pioneering studies in the late 1990s established its application in AIS [4,5]. However, MR-based AIS protocols have limitations and still miss a substantial portion of AIS patients, estimated at 6.8% of total cases in a large meta-analysis [6]. The term DWI-negative stroke has been applied to describe this subgroup of patients. Its prevalence is reported between 5 and 25.6% and it is particularly frequent in patients with milder deficits, consisting of up to 24% of the minor stroke population [6,7,8]. Even so, there is significant heterogeneity among studies of DWI-negative stroke, particularly regarding the timing from symptom onset to first imaging and the follow-up imaging modality [8]. Additionally, data on the relationship between DWI status and endovascular treatment (EVT) remain sparse and the effect of reperfusion has not been completely outlined [9]. A reluctance to establish a stroke diagnosis has been postulated in the presence of a negative DWI scan [10]. Over-reliance on imaging and a lack of understanding of DWI’s limitations, especially by non-neurologists, could lead to the withholding of treatment in false-negative cases, incurring adverse outcomes and increasing disability. Herein, we reviewed the evidence on DWI-negative stroke extensively and delineated the biological, technical, and individual patient factors that predispose to it. We addressed heterogeneity and methodological limitations among included studies, proposing potential future approaches where appropriate. Of note is that most studies referred to DWI-negative strokes before treatment, as interventions were expected to affect the natural course of ischemia; nonetheless, a minority of studies which evaluated DWI status after reperfusion treatment were also included and discussed separately. Overall, the knowledge of when to expect a negative DWI sequence in a patient with AIS is expected to reinforce accurate diagnosis and treatment in clinical practice.

## 2. Biological Interpretation of Restricted Diffusion in Imaging Studies

Diffusion is the result of the Brownian motion of particles in a fluid. In a completely homogeneous liquid, molecules diffuse freely and their movement is random and isotropic. In the brain tissue, the motion of water molecules is limited by cell bodies and axon membranes and they diffuse in an anisotropic manner [11]. Pathological processes alter the distribution of water molecules between tissues [12]. In AIS, metabolic failure caused by ischemia and hypoxia leads to dysfunction of Na^+^/K^+^-ATPase pumps and an ion osmotic gradient that drives the intracellular shift of osmotically active molecules, mainly water particles [13]. This signals the initial step of cytotoxic edema and the first instance of ischemia that can be visualized by DWI within minutes of its onset [5]. Additionally, during DWI acquisition, the apparent diffusion coefficient (ADC) is calculated by an automated process. The ADC value for each brain region represents the degree of restricted diffusion of water molecules in that area. The ADC map sequence is derived by visual presentation of ADC values in every voxel. Inspection of the standard DWI sequence offers qualitative information about diffusion, while the intensity of the signal depression in ADC maps is a quantitative assessment of restriction [12]. Thus, the ability of DWI to capture the initial stages of ischemia and quantitatively assess its severity renders them superior to other sequences.

MRI stroke protocols invariably consist of a combination of sequences; by comparing the features of identified abnormalities, valuable information about lesion age can be obtained. The absence of a visible abnormality in the fluid attenuated inversion recovery (FLAIR) sequence and the simultaneous identification of a lesion in DWI, termed a DWI-FLAIR mismatch, is thought to represent acute injury and tissue at risk that has not yet undergone irreversible ischemia [14]. At the tissue level, the institution of osmotic changes and cytotoxic edema caused by ischemia creates ionic gradients that drive the influx of ions through the endothelial cells into the interstitial space to balance ionic concentrations. As ischemia and ionic edema progress, blood–brain barrier disruption and tight-junction damage support the development of vasogenic edema [13]. Signal intensity augments in FLAIR as tissue water content increases and vasogenic edema develops [15]. The DWI-FLAIR mismatch is used as a tissue-clock marker of ischemic penumbra to guide treatment in cases of unknown time of symptom onset or in patients presenting after awakening with symptoms [14,15]. As infarct age progresses from hours to days, dating can be achieved by examining the appearance of the ischemic lesion on the ADC map. Lesions frequently continue to appear hyperintense on DWI at 30 days; reduced ADC signal intensity in the acute phase progressively increases and transiently matches normal parenchyma, a phenomenon known as ADC pseudonormalization [16]. ADC pseudonormalization is expected at days 5–7 and in some instances up to 3 weeks after infarction; subsequently ADC increases [15,16]. Bright appearance of the infarcted area on the ADC map, concurrent with low signal intensity on DWI due to gliosis, indicates chronic infarction [15]. When follow-up evaluation is performed in the subacute phase, the ADC pseudonormalization phenomenon can potentially lead to clinical misidentification of the event as DWI-negative or averted stroke.

DWI is considered to accurately represent the infarct core. However, the notion that DWI lesions completely match areas of irreversible ischemia has been repeatedly challenged [17,18,19,20]. DWI lesions have been shown to include tissue with low pH that has not yet undergone adenosine triphosphate depletion and thus may be salvageable if reperfusion is achieved [5,17]. The severity of ADC reduction additionally informs about DWI reversibility, with mild ADC decreases associated with tissue salvageability and severe reductions predicting irreversible infarction. Even large volumes of DWI hyperintensities have been reported to reverse after successful revascularization with mechanical thrombectomy [21]. The ischemic core, by definition, indicates tissue destined to infarct; yet, there is selective neuronal loss and differences in cellular responses within the area of ischemia [18]. Gray matter (GM) is more vulnerable than white matter (WM), and GM lesions are less likely to exhibit reversibility of restricted diffusion after reperfusion treatment [18,20]. Furthermore, tissue fate depends greatly on inter-patient variability in susceptibility to ischemia, the degree and duration of hypoperfusion, and the collateral status [5,18,22]. Finally, the volume of DWI abnormality in relation to time from symptom onset has been shown to predict the rate of infarct growth, with greater volumes within the first hours associated with faster progression, lending support to interactions between collateral circulation and DWI lesion morphology [23]. The evidence suggests that DWI visualizes well the early changes taking place during acute infarction, but the appearance and characteristics of the DWI lesion are insufficient to adequately inform about the processes that will lead to eventual tissue necrosis.

Consequently, the limitations of DWI arise from the biological properties of fluid movement within tissues, as well as from the methodological conventions used to investigate these phenomena. Despite progress since the initial conceptualization and application of DWI in clinical practice, the basic mechanisms, such as marked diffusion decrease in acute ischemia and its relation to cell swelling, have not been completely elucidated [11].

## 3. Properties of Diffusion Imaging That Affect Visualization and Sensitivity

Many of the clinically relevant limitations of DWI stem from the technical aspects of the examination. Echo-planar imaging (EPI) is the standard for DWI sequences, allowing for relatively fast scans and reduction in the interference of motion-related artifacts [12]. A standard DWI protocol usually entails scanning in three orthogonal directions with a b-value = 1000–1500 s/mm^2^, producing axial slices of a thickness of 5 mm, with up to 2 mm gap between each slice [5]. The b-value represents a principal parameter of DWI and determines the sensitivity of the sequence to the movement of water molecules. An increase in b-value can be employed to increase sensitivity to hyperacute and small lesions, but such increases also incur signal intensity reduction, an elevated signal-to-noise ratio, and introduce artifacts [24,25]. Furthermore, axial slices with gaps in between them are vulnerable to missing small infarcts and an EPI sequence by itself is prone to susceptibility artifacts at the level of the brainstem. In theory, stronger magnetic fields could result in higher sensitivity by enhancing tissue resolution; however, the effect of the field’s strength remains a matter of debate. Initial reports suggested that the rate of false-negative DWI may be higher in 3 T compared to 1.5 T magnetic resonance scanners when evaluating hyperacute stroke patients [26]. A possible mediator of the latter might be the introduction of susceptibility artifacts in stronger fields. However, subsequent studies did not reproduce this finding and reported a similar or higher sensitivity from the use of 3 T MRI [5,27]. Ract et al. found an increased identification rate of small and brainstem lesions by 3 T MRI when using a 6-direction DWI with a b-value of 2000 s/mm^2^, in comparison to two other 3 T DWI protocols, suggesting that parameter optimization in higher field-strength scanners could lead to better detection of smaller lesions [28].

During image acquisition, the DWI sequence is weighted for diffusion alterations by the use of strong gradient pulses, but inadvertently information from T2 and spin density changes carries over to a variable extent and contributes to the final result, potentially interfering with DWI diagnostic accuracy [29,30]. Most notably, the T2 shine-through effect arises from the high tissue relaxation time necessary to produce diffusion-weighted images, causing processes that exhibit very long T2 decay times to appear as hyperintensities in DWI without a true underlying diffusion restriction. The T2 shine-through phenomenon has also been noted in non-ischemic conditions, such as hypoglycemia, seizures, tumefactive demyelination and drug toxicity [31]. Its presence in stroke mimics may distort the results of studies reporting on DWI-negative AIS prevalence. Close inspection of the signal in the ADC map resolves diagnostic ambiguity in most cases [30,31]. An important impediment to DWI is the impact of eddy-current distortion, which is amplified in magnetic fields of greater strength. Eddy currents lead, among other effects, to geometric distortions and phase shift ghosts (ADC artifacts) and result in errors regarding ADC calculations [30]. Finally, the T2 blackout effect is of less frequent occurrence and pertains to lesions with very short T2 relaxation times that restrict diffusion. Despite true restriction of diffusion, the low T2 signal interferes with DWI signal intensity, leading to lower values and a less bright DWI appearance. This phenomenon is primarily present in cases of intracerebral hematomas [32] and is unlikely to be misinterpreted as DWI-negative AIS.

## 4. Factors Associated with Increased Probability of a Negative Initial DWI

**Figure 1:**
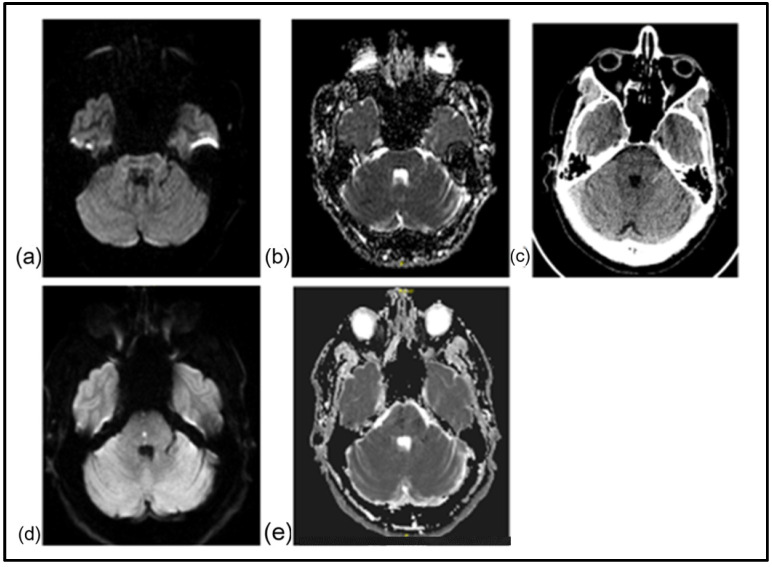
A 49-year-old man presented with dizziness. Clinical examination revealed isolated internuclear opthalmoplegia, indicative of a dorsal pontine lesion. In the initial scan 2 h after symptom initiation, the DWI (**a**) and ADC maps (**b**) were normal. No signs of bleeding were noted in NCCT (**c**) and the patient received intravenous thrombolysis with tenecteplase. Repeat imaging within 24 h revealed a small DWI hyperintensity at the level of the pons (**d**), along with a corresponding decrease in ADC map (**e**) consistent with infarction in the area of the medial longitudinal fasciculus. The patient’s symptoms had improved at discharge (NIHSS score: 1, modified Rankin score: 1) and an excellent outcome with no disability (NIHSS score: 0, mRS: 0) was documented at follow-up 3 months later.

Regarding lesion localization, posterior circulation infarction has been the most frequently identified association with DWI-negative AIS [3,7,33,34,35,36]. A recent meta-analysis estimated the prevalence of DWI-negative AIS at 11–16% overall and recapitulated this finding. Brainstem infarction is tied to the greatest incidence of negative DWI [7,8,37]. From a clinical standpoint, dizziness and internuclear ophthalmoplegia (INO) have been identified as more likely to be accompanied by a normal initial MRI examination [6]. Another study identified isolated vertigo, caudal brainstem syndromes, and focal cortical and lacunar syndromes as presentations of DWI-negative stroke [37]. Cases with acute clinical syndromes indicating a brainstem lesion and a “normal” MRI assessment should raise suspicion for DWI-negative AIS and receive appropriate treatment in an emergency setting (Figure 1). Supratentorial lacunar infarcts and lacunar syndromes such as ataxic hemiparesis also represent predictors of negative acute MRI evaluation [7].

Mild deficits and a low National Institutes of Health Stroke Scale (NIHSS) at presentation are additional important predictors of DWI-negative stroke [5,33]. In the meta-analysis by Alkhiri et al., one out of four patients with minor stroke had a neutral DWI study [8]. In a subgroup analysis of the PRISMS trial, approximately one out of two patients with mild, non-disabling stroke had negative DWI [38]. NIHSS score is a strong predictor of DWI status and has been associated with the risk of positive conversion in initially DWI-negative cases. The incidence of DWI-negative stroke declines as NIHSS score rises [35,39]. However, absence of diffusion abnormalities has also been reported in severe AIS, with NIHSS scores as high as 19 at the time of imaging [40]. The cause may be marked hypoperfusion that produces severe clinical deficits but may be insufficient to establish restriction in diffusion at the time of scan [8,33,37]. These patients must not be missed, as they have been reported to respond to thrombolytic treatment, even in cases of large perfusion deficits [41].

Concerning individual patient characteristics, younger age and female sex have been associated with the probability of DWI-negative infarction, albeit inconsistently [3,8,10,27,38,40,42], as have a history of previous stroke or transient ischemic attack (TIA) [8,10,43]. In a large meta-analysis, ischemic heart disease was more commonly associated with DWI-negative stroke, as opposed to the presence of atrial fibrillation which increased the odds of DWI positivity [8]. In a study of DWI-negative patients with AIS and TIA, smoking and high initial systolic blood pressure were predictive of positive DWI conversion on follow-up imaging [39]. In an Asian cohort, current smoking reduced the likelihood of negative DWI, whereas hypertension and diabetes mellitus did not [40]. In another study in the Asian population, platelet count was correlated with DWI negativity, while hyperlipidemia was not [43]. In studies with greater ethnic/racial heterogeneity, the presence of traditional vascular risk factors, including smoking, did not significantly affect DWI status [8]. Concerning stroke etiology, small-vessel disease and stroke of undetermined cause were associated with DWI negativity whereas cardioembolism was predictive of DWI-positive stroke [35,40]. In a small study of 27 patients with large-vessel occlusion and negative DWI, M2 occlusion was more common than M1 [42].

Timing of imaging from symptom onset affects DWI outcome. Cut-offs reliably predicting DWI-negative AIS continue to be a matter of debate. Earlier works yielded inconsistent results [34,44,45]. Not all earlier studies applied strict timeframes and in some instances, patients who were imaged days after the index event were included. Amongst them, Saur et al. found that DWI status was time independent within 6 h after stroke onset [44]. Bulut et al. found that increases in latency between symptom onset and MRI were associated with positive DWI [46]. In this study, all DWI-negative patients were imaged within 7 h, with a mean time of 4.3 h [46]. Oppenheim et al. [34] demonstrated that the possibility of a DWI-negative vertebrobasilar stroke was greater on the first day and then diminished significantly. A low signal-to-noise ratio in the first hours of infratentorial infarction enhances this effect [34]. Additionally, posterior circulation infarcts exhibit delayed visualization of DWI abnormalities when compared to anterior circulation [27]. A meta-analysis including studies up to 2015 suggested a potential increase in DWI-negative strokes when DWI was performed within 3 to 6 h from symptom onset [6]. In a more recent study, the majority of DWI-negative AIS patients were imaged within 6 h, with a median time of 5 h [37]. Conversely, there are studies that have reported DWI-negative patients scanned at later time windows compared to those with DWI-identifiable lesions [3,10]. Overall, MRI performed in the hyperacute phase of an ischemic event appears to be linked with increased odds of DWI negativity: for every hour elapsing from symptom onset to imaging, the probability for a positive DWI increases by 4% [35].

## 5. Safety and Efficacy of Acute Reperfusion Therapies in DWI-Negative Stroke

Absence of a DWI lesion prior to reperfusion treatment has been associated with better outcomes and a lower risk of intracerebral hemorrhage [8,36,40,42]. In their meta-analysis, Alkhiri et al. [8] found markedly lower rates of symptomatic intracerebral hemorrhage (sICH). All types of stroke were included, but the study was biased towards patients with minor deficits [8]. In a study directly examining the association between DWI negativity and treatment effect in posterior circulation stroke, a greater proportion of the 100 DWI-negative AIS patients treated with tenecteplase had excellent functional outcome and lower overall mortality compared to the control group [47]. Furthermore, Zhu et al. performed MRI within 72 h after intravenous thrombolysis (IVT) and reported a statistically significant reduction in hospitalization days and a better functional outcome in patients with negative DWI [36]. Accordingly, in a recent study, DWI-negative stroke patients treated with IVT experienced less early neurological deterioration compared to those with a positive DWI [43]. Finally, in a small study of wake-up strokes, the DWI-negative group experienced no hemorrhages after IVT, had a lower mRS score, and showed a trend towards favorable functional outcome at discharge [48].

**Figure 2:**
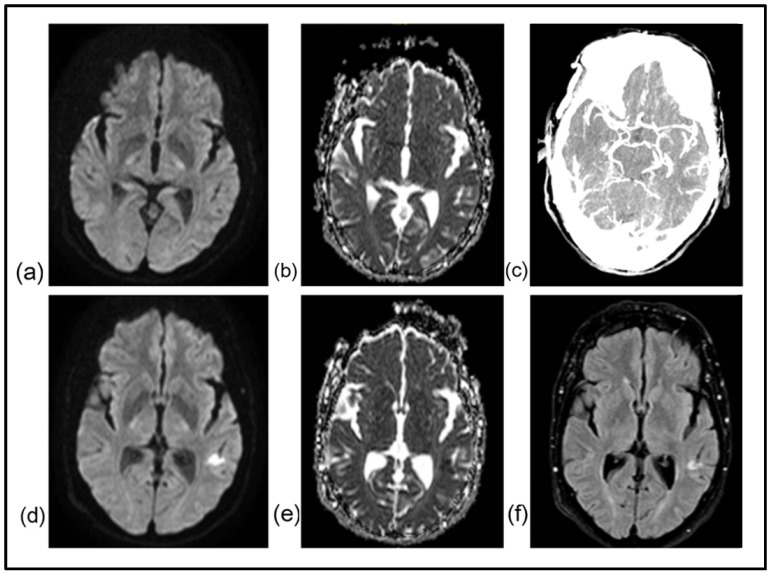
A 68-year-old woman with a history of hypertension and hypothyroidism awoke from her afternoon sleep with right-sided weakness and difficulty in speaking. She then experienced a transient improvement without full resolution of symptoms and then deteriorated. She arrived in our hospital 6 h after symptom onset. Neurological examination revealed aphasia, right-sided facial droop, and right arm weakness (NIHSS score: 9). Emergent MRI revealed no signs of lesion in DWI (**a**) and ADC map (**b**). CT angiography ruled out the presence of LVO and the peripheral MCA branches showed adequate filling (**c**). Based on the absence of bleeding and a neutral MRI scan, she was treated as a DWI-negative AIS and received IVT with tenecteplase. Early improvement was noted and subsequent MRI evaluation 20 h after the first scan revealed an infarct in the distribution of the left MCA (**d**–**f**). She was discharged after a short hospitalization, with minimal disability (NIHSS score: 1, mRS: 1). Work-up led to the diagnosis of atrial fibrillation. Initial symptom fluctuation suggests a potential degree of spontaneous reperfusion and improvement after IVT indicates successful recanalization.

Interestingly, the degree of signal reduction in ADC maps is a reliable predictor of hemorrhagic transformation following acute reperfusion treatment [5]. Absence of findings in the DWI of a patient with acute neurological deficits may cause reluctance to treat [10]. Suspicion of a stroke mimic may lead to inappropriate delays or omissions in treatment, leading to potentially harmful effects. Yet, these patients exhibit low hemorrhagic risk and better odds for good prognosis when treated with IVT, including individuals who present in the extended time window (Figure 2). Limited data suggest that DWI-negative AIS patients are not treated less than their DWI-positive counterparts. A systematic review of 12,018 AIS patients demonstrated higher percentages of IVT in the DWI-negative group (16.31% vs. 8.82%) [40]. These patients tended to be younger and presented with lower NIHSS scores, possibly leading treating physicians to perceive them as ideal candidates for IVT.

We did not identify any studies focusing on the characteristics of DWI-negative patients who were treated with MT. Two of the studies that demonstrated a positive association between negative initial DWI and good outcomes after treatment included MT patients [42,48]. The total number of 20 patients is too small to allow generalization of the findings. Overall, DWI volume before MT, as well as 24 h after intervention, has been associated with prognosis, with greater volumes inversely correlating to favorable outcomes [47]. Regarding treatment effect, Launhardt et al. [49] performed thrombectomy in 28 patients with basilar artery occlusion, and 5 exhibited DWI lesion reversal following successful reperfusion, which was associated with an increased rate of good clinical outcome. They found that DWI reversibility was present only in brainstem infarcts and suggested that this phenomenon might not be uncommon in this brain region [49].

Spontaneous reperfusion, reperfusion following treatment and the reversal of DWI phenomenon (DWIR), may affect the prevalence of DWI-negative AIS. DWIR refers to decreases in the number of hyperintense DWI voxels or in lesion volume in the follow-up MRI of treated AIS patients. It is usually partial and only 0.8% of lesions were reported to exhibit complete and sustained reversal prior to widespread use of MT [50,51]. DWIR is estimated to take place in approximately 20–25% of AISs and is associated with spontaneous recanalization, reperfusion treatments, earlier initiation of IVT and MT, is more frequent in small lesion volumes but can also be present in volumes >70 mL, and represents a reiteration of the observation that in hyperacute infarction, DWI volume does not entirely equate to the ischemic core [17,51,52,53,54]. Skattor et al. investigated 303 patients who underwent MT and found a greater extent of DWIR in the WM, no difference in likelihood of reversal between cortical and WM regions, and a persistence of DWIR after one month in 47% of patients with initial reversal [20]. The magnitude of MT effect may be greater than that of IVT and this has been demonstrated even in AISs with large infarct cores [20,53]. In an evaluation of 211 MT-treated patients with DWI-ASPECTS 0-5, successful reperfusion predicted DWIR and DWIR exhibited statistically significant association with early neurological improvement and clinical outcomes at 3 months [9].

Spontaneous recanalization has been reported to occur in 20–25% of cases of acute stroke [37,55,56]. Early spontaneous recanalization has been postulated as a mechanism of DWI negativity [7]. Recanalization rates increase with the use of reperfusion treatments during the acute phase [57]. Patients who are scanned very early after symptom onset have elevated odds of a normal DWI. Li et al. enrolled 437 stroke patients who received IVT within 4.5 h; 54 patients (12.36%) had negative post-IVT DWI [58]. A recent study included 151 AIS patients; 23.2% had negative DWI post-IVT and an association with posterior circulation infarction and lower NIHSS scores was demonstrated. Notably, deficits in the initial CT perfusion (CTP) were found in 37% of the post-IVT DWI-negative group [36]. Additionally, shorter time between symptom onset and treatment administration, as well as longer duration to MRI scan after IVT, have been associated with increased odds of DWI negativity [43,47].

**Figure 3:**
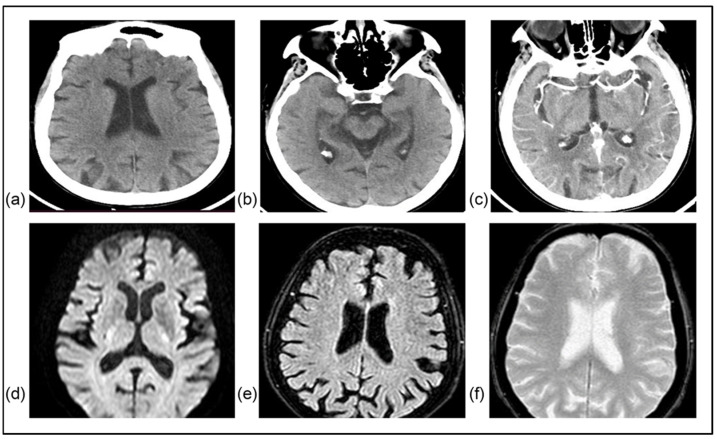
A 83-year-old woman with a medical history of hypertension, end-stage renal disease, and breast cancer in remission presented with right hemiparesis 3 h after symptom onset (NIHSS score: 6, mRS: 4). Initial NCCT (**a**,**b**) and CT angiography (CTA) (**c**) were unrevealing of parenchymal damage or large-vessel occlusion. She received IVT with alteplase within 30 min of arrival and experienced complete symptom resolution within the first 20 h. In MRI 9 days later, no signal hyperintensities were noted in DWI (**d**) and FLAIR (**e**) sequences. In T2* imaging (**f**), signal depression indicating bleeding or the presence of thrombus within a vessel was not identified. Thorough investigation during hospitalization did not reveal any likely cause of stroke mimic. This case represents an averted stroke: complete symptom resolution following reperfusion treatment, with negative subsequent imaging.

Cases of neurological deficit resolution and negative MRI evaluation following reperfusion treatment (IVT or MT) are referred to as averted or aborted strokes (Figure 3). Predisposing factors for averted stroke and DWI-negative AIS significantly overlap; low NIHSS, younger age, female sex, and no evident LVO are associated with both [8,10,22]. Importantly, earlier IVT was linked with better odds of early symptom resolution but not with tissue-defined aversion of stroke [22]. Averted stroke may form a non-trivial proportion of DWI-negative AIS cases. A review of 8101 DWI-negative AIS syndromes reported that aborted stroke comprised 5.2% of all cases [37]. Symptom resolution within 24 h is frequent after reperfusion treatment in this patient population. An earlier study comprising 254 IVT-treated patients classified 3.5% of the cases as stroke mimics and 9.1% of them as TIAs based on normal DWI on subsequent assessment. The authors suggested that the term averted stroke might have better fit the description of the TIA group, owing to the likelihood that the natural course of the event was altered by treatment [59].

## 6. Discussion

Our comprehensive review identified rates of DWI-negative stroke as ranging widely from 6.6% to 23.2% in studies including various lesion localizations; however, posterior circulation ischemia is associated with higher rates of DWI negativity. The majority of these studies were based on single-center cohorts mainly in Asian populations, potentially introducing biases in the reported prevalence and predisposing factors (Table 1). Stroke etiology, pathophysiology, and the impact of risk factors vary among ethnic groups. A higher proportion of lacunar infarcts and small-vessel disease in Chinese patients has been suggested in comparison to Caucasian populations [60], supporting a trend towards greater DWI-negative stroke prevalence estimation. Furthermore, increased incidence of intracranial arterial stenosis and greater association of systolic and diastolic blood pressure with stroke have been demonstrated in Asian populations [61,62]. The latter might account for variability regarding the relationship between vascular risk factors and DWI-negative stroke [8,39] Future research on DWI-negative stroke should address this issue and aim to include homogeneous populations or focus on under-reported ethnic groups.

The relationship between the timing of the scan and symptom onset remains a matter of debate. Nonetheless, large studies support a link between DWI-negative AIS and imaging within the first 6 h; an increase in the rate of DWI positivity for every hour elapsing from symptom onset suggests that hyperacute scans, combined with other predisposing factors, elevate the probability of negative DWI [5,8,35,37,39,46]. Conversely, an increase in time from IVT administration to MRI completion has been associated with DWI negativity [47], likely owing to the longer duration available to the thrombolytic agent to exert its effect. Initially, DWI-negative AIS may often exhibit an identifiable lesion in later scans, as did 22.5% of patients in the series by Kim et al. [39]. There is temporal evolution of ADC values in the acute stage and DWI volume progression exhibits significant variability among patients, reaching maximum values within 13 to 247 h from onset depending on individual characteristics [16,63]. The site and size of the infarction, collateral status, and tissue susceptibility to ischemia also influence the temporal evolution on imaging, with pathophysiological and technical factors predisposing delayed visualization of posterior circulation lesions [18,27,34,64,65]. Thus, serial MR imaging has demonstrated that early DWI, especially in the first hours of the event, is expected to increase the rates of DWI-negative AIS and that the latter might often more aptly be called “DWI-negative AIS at the time of imaging”.

Many institutions have added coronal and sagittal diffusion imaging to their MRI protocols to enhance sensitivity, especially in cases of brainstem stroke. Coronal DWI performs excellently in identifying midbrain lesions and the combination of axial and coronal DWI had perfect accuracy in a study of 134 brainstem AIS [66]. In another study, the application of thin 3 mm axial slices increased sensitivity to 100% in the detection of infratentorial lesions; however, the addition of 3 mm coronal slices did not improve diagnostic performance [67]. One out of three DWI-negative AIS patients may harbor underlying perfusion deficits and the inclusion of perfusion-weighted imaging (PWI) increases the sensitivity of stroke detection [3,36]. The ensuing increase in scanning time is acceptable to avoid missing potentially treatable strokes; yet, 2 to 3 of 100 patients escape detection by the combined use of DWI and PWI. Moreover, perfusion imaging also has limitations, including in posterior circulation and lacunar stroke and is more susceptible to motion artifacts [65]. Additionally, LVO stroke patients with negative diffusion and perfusion imaging have been reported. Meticulous inspection of the FLAIR sequence may obviate the need for additional perfusion imaging. In the setting of large-vessel occlusion, thrombus visualization on FLAIR was present in 68.1% of DWI-negative AIS patients and the “spaghetti sign”, caused by slow flow, was present in 83% of DWI-negative cases [42]. Collateral status, especially in LVO, can affect the extent and the rate of progression of ischemia impacting DWI status [5,64,68]. Collateral status is associated with DWI lesion volume and collateral profile can inform about treatment effect and prognosis [64,68]. In practice, good collateral status in addition to the concurrent presence of the “FLAIR spaghetti sign” or the “SWI brush sign” should raise suspicion for a DWI-negative LVO stroke. Moreover, intermediate or good collaterals as assessed by CTA are associated with smaller DWI lesions post-MT [64], substantiating the possibility of complete reversal after intervention in a minority of cases.

Processing algorithms capable of extracting information about perfusion status from DWI sequences based on intravoxel incoherent motion are presently in development and are limited to research purposes [69]. Furthermore, the application of Artificial Intelligence (AI) software promises faster and more accurate evaluation and processing of DWI. Deep learning-based AI applications have performed adequately in testing scenarios, with an accuracy of 95% for identifying stroke patients and a sensitivity of 83.5% to DWI lesions [70]. A two-stage deep learning externally validated module correctly identified patients with an unknown time of onset within the 4.5 h therapeutic window based on DWI–FLAIR mismatch [71]. Nevertheless, the AI model missed smaller lesions and infarcts with lower ADC signal or minimal slice coverage, similarly to human-read DWI scans [70]. Likewise, automated ADC thresholding software did not consistently match manual delineation performed by an expert and was unreliable for abnormalities of very small size [19]. The inherent variability in ADC lesion values between patients and the influence of parenchymal ADC were important factors contributing to subpar performance by the automated tool [19]. Overall, software with clinical implications needs further testing in prospective trials focused on outcomes before routine implementation [70].

Correctly identifying an acute cerebrovascular event despite a negative DWI study has important prognostic and decision-making value. In comparison to DWI-positive AIS, DWI-negative patients had better odds of good outcomes at discharge and 3-month follow-up and lower rates of stroke recurrence, combined vascular events, severe disability, and mortality [8,40]. Additionally, DWI-negative AIS patients are good candidates for acute reperfusion treatments, with lower rates of hemorrhagic complications and favorable functional outcomes compared to their DWI-positive counterparts [8,36,41,43,47,48,58]. Furthermore, conversion to positive DWI on follow-up after an initial negative scan has been associated with early neurological deterioration and a greater risk of stroke recurrence [39]. Kim et al. have devised the predictive DWI-CONVERSION score which uses factors such as the presence of atrial fibrillation, objective hemiparesis, high blood pressure, and cholesterol at presentation, as well as NIHSS and symptom duration, to identify patients at risk of delayed conversion [39]. Interestingly, studies following DWI-negative patients for longer periods found that lower stroke recurrence risk and better functional outcomes were not sustained 1 year after the index event [8]. It is likely that DWI negativity contributes differently depending on disease duration and that other factors may exert a greater impact than DWI status in later stages.

The phenomenon of DWIR has been reported in 26.5% of ischemic strokes and acute reperfusion treatments are a major contributor to it [20,51,52]. A proportion of patients with small infarct volumes are expected to transiently reverse to a volume close to zero. Minute hyperintensities may be missed and cases erroneously marked as DWI-negative AIS might be included in studies, leading to an inflated prevalence. Instances of persistent and complete lesion reversal are rare and may be better classified as averted strokes [22,50]. A minority of studies on DWI-negative AIS elected to enroll patients after treatment administration without prior MRI [27,36,43,47]. This approach is problematic because it confounds factors predisposing DWI negativity with treatment effect. We decided not to focus on factors predisposing DWI negativity derived from post-IVT AIS studies. Future systematic reviews investigating predictors of DWI-negative stroke had better follow a similar approach to reduce bias. Treatment alters the natural progression of infarction and in the case of early symptom resolution, differentiation between stroke and TIA may be difficult. This variability in the description of DWI-negative AIS hinders systematic analysis of the collected data and underscores the need for a comprehensive, widely accepted definition. Furthermore, tenecteplase has been introduced in recent years as an alternative to alteplase and has been shown to act more rapidly and achieve higher rates of complete recanalization [72,73]. Thus, studies using different thrombolytic agents should not be combined in meta-analyses of DWI-negative AIS due to potentially substantial heterogeneity in treatment effects.

Studies that do not incorporate follow-up MRI after a DWI-negative scan are at increased risk of including stroke mimics [74]. For instance, transient cytotoxic edema with restricted diffusion may occur in the post-ictal state due to seizure-related metabolic exhaustion and is typically reversible, thus being distinguishable from irreversible ischemic injury [75]. On the other hand, stroke chameleons are events with uncommon AIS symptoms, which are easy to misidentify under the circumstances of a negative initial DWI. Stroke chameleons share predisposing factors with DWI-negative AIS, notably infratentorial location, younger age, lower blood pressure values at presentation, undetermined etiology, and lower NIHSS scores [76]. DWI-negative stroke chameleons are expected to be under-represented in relevant studies and may potentially lead to prevalence underestimation. Finally, the way DWI-negative AIS is currently defined can, by itself, be a source of confusion. A subgroup analysis of the WAKE-UP trial focusing on DWIR reported the exclusion of 4 patients with a DWI lesion volume of 0 mL, even though the lowest DWI volume in WAKE-UP was 0.8 mL and there were no DWI-negative cases among the 503 enrolled patients [51,77]. Consequently, future studies on DWI-negative AIS should use stricter definitions and multiple follow-up imaging evaluations to reduce erroneous inclusions.

The case descriptions herein highlight how confidence in diagnosing acute stroke in the absence of DWI findings guides appropriate management and underscore that despite constant advances in imaging, stroke remains first and foremost a clinical diagnosis. In our first case, a young adult presented hyperacutely with dizziness and signs of INO, a phenotype associated with increased likelihood of negative initial DWI. The second patient sought medical help after symptom fluctuation suggesting spontaneous recanalization followed by subsequent reocclusion. The important clinical deficits, moderately high NIHSS, increased time to imaging, and cardioembolic cause would favor DWI positivity in the absence of a counteracting mechanism. Indeed, there was DWI conversion in the post-IVT MRI 24 h later, with a substantial DWI lesion. Finally, we included a case of averted stroke following treatment with IVT in a presumed etiological background of small-vessel disease. Patients with averted stroke should not be misidentified as stroke mimics; however, diagnosing stroke in the context of symptom resolution and negative imaging studies is demanding. Understanding biological and epidemiological factors that predispose DWI negativity offers additional guidance. These cases highlight the integral role of stroke neurologists in the treatment of DWI-negative AIS.

## 7. Conclusions

Earlier studies considered the phenomenon of DWI-negative AIS as a false-negative. However, a negative DWI may be an expected finding in specific instances of hyperacute stroke. The application of advanced diffusion imaging modalities for acute stroke evaluation provides enhanced sensitivity, but is not bereft of limitations and blind spots stemming from the biological correlates of diffusion imaging and the technical aspects of the examination. In a statement paper, the American Academy of Neurology recommended using DWI for acute stroke diagnosis owing to its superiority, but noted that it is expected to be insufficient in specific patient populations [78]. Awareness about predisposing factors, interactions with treatment, and association with prognosis guides appropriate decision-making. Future research should clarify a DWI-negative AIS definition and its prevalence and predisposing factors by inclusion of different ethnical populations and onset-to-imaging time intervals.

## Figures and Tables

**Table 1:** Studies reporting on DWI-negative AIS prevalence since 2021.

Study	DWI-Negative AIS Prevalence	Description/Limitations
Pektezel et al., 2021 [37]	6.6% 8101 cases, 535 DWI-negative	Combination of a single-center cohort of 1506 patients [DWI-negative n = 20, (1.3%)] with cases from the literature. Increased heterogeneity and potential underestimation.
Kim et al., 2021 [39]	13.2% 5271 cases, 694 DWI-negative	Retrospective single-center study that included TIA patients. Potential overestimation.
Wang, Yu et al., 2022 [40]	7.7% 12,026 cases, 932 DWI-negative	Prospective multi-center cohort study, included a relatively low number of non-minor (NIHSS > 5) AIS patients.
Zhu et al., 2023 [36]	23.2% 151 cases, 35 DWI-negative	Retrospective single-center study, smaller sample size compared to the other studies. DWI status was evaluated post-IVT.
Fotso et al., 2024 [42]	3.9% 1210 cases, 47 DWI-negative	Retrospective single-center study. Only patients with anterior circulation AIS were enrolled.
Li et al., 2025 [43]	22.74% 277 cases, 63 DWI-negative	Single-center study. Included only posterior circulation strokes. DWI status was evaluated post-IVT. Small sample size and missing clinical data in a small number of patients, variability in follow-up may have introduced biases and the risk of stroke mimic inclusion.
Alkhiri et al., 2024 [8]	16%, 11% after sensitivity analysis. 16,268 cases, 2603 DWI-negative	Meta-analysis. Significant heterogeneity between studies, I^2^ = 91% estimated by the authors after sensitivity analysis. Temporal heterogeneity, the earliest study was conducted in 2000 and the most recent in 2023. Due to potential over-representation of minor stroke patients, sensitivity analysis was performed.

## Data Availability

The original contributions presented in this study are included in the article. Further inquiries can be directed to the corresponding author.

## Abbreviations

The following abbreviations are used in this manuscript:

DWI	Diffusion-weighted imaging
AIS	Acute ischemic stroke
DWI-negative	DWI-negative stroke
IVT	Intravenous thrombolysis
MT	Mechanical thrombectomy
NCCT	Non-contrast computed tomography
MR	Magnetic resonance
ADC	Apparent diffusion coefficient
FLAIR	Fluid attenuated inversion recovery
BBB	Blood–brain barrier
GM	Gray matter
WM	White matter
EPI	Echo-planar imaging
SNR	Signal-to-noise
T	Tesla
INO	Intranuclear ophthalmoplegia
NIHSS	National Institutes of Health Stroke Scale
TIA	Transient ischemic stroke
LVO	Large-vessel occlusion
DWIR	DWI-reversal
ICH	Intracerebral hemorrhage
ASPECTS	Alberta Stroke Program early CT score
CTP	Computed tomography perfusion
CTA	Computed tomography angiography
SWI	Susceptibility-weighted imaging
PWI	Perfusion-weighted imaging
ASL	Arterial spin labeling
AI	Artificial intelligence
Afib	Atrial fibrillation
